# Supplementary material for: Low contraceptive utilization among young married women is associated with perceived social norms and belief in contraceptive myths in rural Ethiopia
Source: PLoS One. 2021 Feb 22;16(2):e0247484. doi: 10.1371/journal.pone.0247484 (PMC7899365; doi:10.1371/journal.pone.0247484)
Supplement: S1 Table — (DOCX) [file pone.0247484.s001.docx]

**S1 Table. Frequency of each item used for measuring perceived social approval and belief in contraceptive myths among young married women in Kersa HDSS, Eastern Ethiopia, 2018**

| **Items** | **Frequency (%)** | |
| --- | --- | --- |
| **Items used to measure injunctive norms** | **Yes** | **No** |
| My parents believe I should use birth control to prevent pregnancy : Strongly disagree  Disagree  Neutral  Agree  Strongly agree | 53(14.5)  80(21.9)  21(5.7)  176(48.1)  36(9.8) | 783(29.7)  827(31.4)  307(11.7)  628(23.8)  89(3.8) |
| Most of my friends believe using birth control is important: Strongly disagree  Disagree  Neutral  Agree  Strongly agree | 31(8.4)  70(19.0)  77(20.9)  152(41.3)  38(10.3) | 564(21.4)  761(28.9)  615(23.4)  614(23.3)  78(3.0) |
| My husband believes use of contraceptive method is good to avoid pregnancy Strongly disagree  Disagree  Neutral  Agree  Strongly agree | 1694.4)  52(14.3)  25(6.9)  232(63.6)  40(11.0) | 575(21.9)  891(33.9)  271(10.3)  782(29.8)  106(4.0) |
| My mother in-law believe I should use contraceptive: Strongly disagree  Disagree  Neutral  Agree  Strongly agree | 52(14.2)  81(22.1)  67(18.3)  155(42.4)  11(3.0) | 485(32.2)  915(34.9)  386(14.7)  425(16.2)  53(2.0) |
| **Items used measure believes in contraceptive myths and misconception** |  |  |
| Contraceptives are dangerous to women’s health: Strongly disagree  Disagree  Neutral  Agree  Strongly agree | 121(33.0)  195(53.1)  6(1.6)  41(11.2)  4(1.1) | 779(29.5)  1092(41.4)  228(8.7)  412(15.6)  126(4.8) |
| Contraceptives can harm women’s womb: Strongly disagree  Disagree  Neutral  Agree  Strongly agree | 77(21.1)  142(38.9)  16(4.4)  107(29.3)  23(6.3) | 428(16.3)  680(25.9)  268(10.2)  911(34.6)  344(13.1) |
| Contraception would ruin sexual mood: Strongly disagree  Disagree  Neutral  Agree  Strongly agree | 141(38.6)  138(37.8)  50(13.7)  29(8.0)  7(1.9) | 725(27.6)  840(31.9)  659(26.4)  280(10.6)  92(3.5) |
| Use of a contraceptive can make a woman permanently infertile: Strongly disagree  Disagree  Neutral  Agree  Strongly agree | 121(33.2)  133(36.4)  32(8.8)  69(18.9)  10(2.7) | 701(26.6)  727(27.6)  349(13.3)  662(25.2)  192(7.3) |
| Contraceptives can give you deformed babies: Strongly disagree  Disagree  Neutral  Agree  Strongly agree | 96(26.2)  126(34.4)  27(7.4)  101(27.6)  16(4.4) | 580(22.0)  610(23.2)  366(13.9)  807(30.7)  268(10.2) |
| Women who use contraceptive methods may become promiscuous: Strongly disagree  Disagree  Neutral  Agree  Strongly agree | 205(56.3)  126(34.62)  14(3.9)  18(5.0)  1(0.3) | 1377(52.5)  809(30.8)  221(8.4)  155(5.9)  63(2.4) |
