# Supplementary material for: Low contraceptive utilization among young married women is associated with perceived social norms and belief in contraceptive myths in rural Ethiopia
Source: PLoS One. 2021 Feb 22;16(2):e0247484. doi: 10.1371/journal.pone.0247484 (PMC7899365; doi:10.1371/journal.pone.0247484)
Supplement: S2 Table — (DOCX) [file pone.0247484.s002.docx]

**S2 Table. Frequency of each item used to assess knowledge of contraceptive methods among young married women in Kersa HDSS, Eastern Ethiopia, 2018**

| **Knowledge of contraception** | | Frequency | Percentage |
| --- | --- | --- | --- |
| Oral contraceptive pill | Yes  No | 1782  1257 | 58.6  41.4 |
| Woman cannot to get pregnant for at least two months after stop taking birth control pills | Yes  No | 422  2617 | 13.9  86.1 |
| If a woman is having side effect with one kind of pill, switching to another type or brand might help. | Yes  No | 928  2,111 | 30.5  69.5 |
| Injectable | Yes  No | 2,487  546 | 82.0  18.0 |
| Even if a woman is late getting her Depo-Provera (Injection), she is still protected from pregnancy for at least 3 months. | Yes  No | 539  2,500 | 17.7  82.3 |
| Implants | Yes  No | 2,338  702 | 76.9  23.1 |
| Long-acting methods like the implants can be removed early, if a woman want to get pregnant | Yes  No | 2,773  266 | 91.3  8.7 |
| Intrauterine contraceptive device (IUCD) | Yes  No | 150  2,896 | 4.9  95.1 |
| Emergency Contraception | Yes  No | 52  2,987 | 1.7  98.3 |
| Male condom | Yes  No | 379  2,660 | 12.5  87.5 |
| Female sterilization | Yes  No | 534  2,490 | 17.7  82.3 |
| Lactational Amenorrhea Method (LAM) | Yes  No | 1072  1,964 | 35.3  64.7 |
| Rhythm Method | Yes  No | 184  2,833 | 6.1  93.9 |
| A woman who is still breast feeding can get pregnant | Yes  No | 1059  1,978 | 34.9  65.1 |
| A woman can get pregnant before she has her first period after gave birth | Yes  No | 1,522  1,512 | 50.2  49.8 |
