# Supplementary material for: Low contraceptive utilization among young married women is associated with perceived social norms and belief in contraceptive myths in rural Ethiopia
Source: PLoS One. 2021 Feb 22;16(2):e0247484. doi: 10.1371/journal.pone.0247484 (PMC7899365; doi:10.1371/journal.pone.0247484)
Supplement: Attachment — Submitted filename: PONE-D-20-05351.docx [file pone.0247484.s004.docx]

**Low contraceptive utilization among young married women is associated with perceived social norms and believes in contraceptive myths in rural Ethiopia**

Thank you for the opportunity to review this paper. The authors examined the influence of perceived social norms and beliefs about contraception on contraceptive use among married adolescents and young women in Ethiopia. Data is obtained from a community survey of 3039 young married women aged 14-24 years. A multivariable logistic regression analysis was used to identify factors associated with contraceptive use.

**General comment**

The topic is a very interesting one that is relevant to family planning policies and programmes. There is a growing body of evidence on the link between perceived social norms/contraceptive beliefs and contraceptive use. Although there is merit in the contribution to knowledge, the paper has some conceptual flaws in the analysis and presentation of the results. Authors included in their analysis of the effects of beliefs on contraceptive use, participants who are not aware of any contraceptive method. This introduces biases in the results. It is not possible to evaluate someone's beliefs and use of contraceptives when they are not aware of it. Analysis of the link between beliefs and contraceptive use should be restricted to those who have ever heard of contraception. It is not clear what study design was used. The study cannot be generalized given its weak design. There are a lot of grammatical errors that should be corrected by a language expert for it to meet publication standards.

**Specific comments**

**Title**

The title of the paper should be written in a proper way highlighting its core content. Currently, it sounds more like a conclusion of the study rather than a title. It should be revised!. The title includes the text‘…in rural Ethiopia’ yet the sample includes participants from urban settings.

**Abstract**

There is a lack of clarity on how the association between outcome and independent variables are reported. Report only factors associated with contraceptive use.

**Introduction**

Outlining a conceptual model would be useful. The authors should also highlight key hypotheses to be tested in the study.

**Methods:**

It is not clear why the study computed a sample size of 1566, yet all 3102 young women in the study area were interviewed. It does not add value to calculate the sample size when a census is used. There need to clarify why all young women were interviewed instead of the number that was sampled.

The inclusion of place of residence, rural vs. urban, is problematic given that the urban sample size is negligible. The result is greatly biased given that the sample was not stratified to allow for comparison between rural and urban.

There is a high correlation between age and number of children. Also, adolescents <19 years are more vulnerable and are not as exposed compared to young women 20-24. The study can be strengthened by comparing participants <19 years with those aged 20-24years

**Result**

**Table 2:** Instead of bivariate logistic regression, do a simple comparison between contraceptive users and non-users by perceived social approval and beliefs using column totals. It also important to compare the mean scores of index indicators between users and non-users

**Table 3:** The title is ‘Multivariate logistic regression…’ However, there are some bivariate results in columns 2&3. These should move to Table 2.

It is not clear which variables are controlled in the multivariate model. This makes it very difficult to infer the effects of perceived social norms and beliefs on contraceptive use. A more precise way to handle this would to running a separate model with the main independent variables without controls and another with the controls.

Review how the logistic regression results are reported. There seems to a confusion between the outcome and independent variables in the way the odds are reported. It is, therefore, very difficult to understand the magnitude and the direction of the effect of the independent factors on the outcome.
