# Supplementary material for: Low contraceptive utilization among young married women is associated with perceived social norms and belief in contraceptive myths in rural Ethiopia
Source: PLoS One. 2021 Feb 22;16(2):e0247484. doi: 10.1371/journal.pone.0247484 (PMC7899365; doi:10.1371/journal.pone.0247484)
Supplement: Attachment — Submitted filename: Response to Reviewers.docx [file pone.0247484.s005.docx]

29 June 2020

To: PLOS ONE Academic Editor,

Manuscript ID No. PONE-D-20-05351

**Subject: Response to academic editor and reviewers comment**

Please find response to each point raised by the academic editor and reviewers to our manuscript entitled “Low contraceptive utilization among young married women is associated with perceived social norms and believing in contraceptive myths in eastern Ethiopia”, which we would like to resubmit for publication as an original article in PLOS ONE.

Your comments and those of the reviewers were highly insightful and enabled us to greatly improve the quality of our manuscript. Our point-by-point response to each of the comments of the reviewers as well as your comments is attached with this letter. We hope that the revisions in the manuscript and our accompanying responses will be sufficient to make our manuscript suitable for publication in the PLOS ONE.

We shall look forward to hearing from you at your earliest convenience.

Yours sincerely,

**Response to Academic and Editor and Reviewers comment of the Manuscript entitled “*Low contraceptive utilization among young married women is associated with perceived social norms and believes in contraceptive myths in eastern Ethiopia*”**

| **Reviewer concern/Recommendation** | **Authors Response** | **Section modified** |
| --- | --- | --- |
| **Reviewer 1** | | |
| The title of the paper should be written in a proper way highlighting its core content. Currently, it sounds more like a conclusion of the study rather than a title. It should be revised! The title includes the text‘…in rural Ethiopia’ yet the sample includes participants from urban settings. | Thank you for your point! We indicated as ‘among rural’ since the proportion of urban residents is less than 8%. Now, we have removed the word rural in the revised manuscript based on your suggestion, but we have found the other part of the title is descriptive and conveys the key finding of the study. | Title |
| Abstract: There is a lack of clarity on how the association between outcome and independent variables are reported. Report only factors associated with contraceptive use. | We have revised it accordingly. | Abstract, line 46-53 |
| Introduction: Outlining a conceptual model would be useful. The authors should also highlight key hypotheses to be tested in the study. | That is an interesting suggestion. We have modified the introduction section based on your suggestion. | 82-110 |
| It is not clear why the study computed a sample size of 1566, yet all 3102 young women in the study area were interviewed. It does not add value to calculate the sample size when a census is used.  There need to clarify why all young women were interviewed instead of the number that was sampled. | The sample size calculation was indicated in the manuscript aiming to communicate the minimum sample size required was achieved. Now, we have revised the section since we understand that it may confuse readers. | Study population, line 131-140 |
| The inclusion of place of residence, rural vs. urban, is problematic given that the urban sample size is negligible. The result is greatly biased given that the sample was not stratified to allow for comparison between rural and urban. | As indicated in the methods, all the target population in demographic and health surveillance site was included in the study. Since we included all the target population in study area, it was not required to employ either stratification or any other sampling method. |  |
| There is a high correlation between age and number of children that might affect the results when these two variables are included in the regression model. | We conducted a multicollinearity analysis to evaluate the association between the two independent variables and only age was included in the multivariate analysis regression model since the correlation coefficient was >=0.5. We selected age since the question about the number of children was skipped for more than one-fourth of the study participant never gave birth which significantly reduced the sample size and also disturbed the model goodness of fit. |  |
| Also, adolescents <19 years are more vulnerable and are not as exposed compared to young women 20-24. The study can be strengthened by comparing participants <19 years with those aged 20-24 years | Thank you for this essential suggestion! We have taken into account and added the result that compares contraceptive utilization among adolescent and young adult women in the result section and fig1. The association of age with contraceptive utilization is also indicated in the multivariate regress analysis. | Result section,  line 295, 296, and on fig.1 |
| Table 2: Instead of bi-variate logistic regression, do a simple comparison between contraceptive users and non-users by perceived social approval and beliefs using column totals. It also important to compare the mean scores of index indicators between users and non-users | Good point. We have revised table 2 and included it as a supporting information in the revised manuscript. Mean scores of index indicators between users and non-users were also indicated in the table 2 of the revised manuscript. | Result,  table 2 (line 314) and S1 Table |
| Table 3: The title is ‘Multivariate logistic regression…’ However, there are some bi-variate results in columns 2 & 3. These should move to Table 2. | The aim of table3 was to show the outcome of multivariate logistic regression analysis. However, we have added the 2nd & 3rd column assuming it benefits future researchers to get information on the proportion/mean of explanatory variables among outcome. Column 3 can also help to easily evaluate how much the effect size of each variable changed after adjusting for other covariates. Now, we have modified the title of the table. | Result section,  Table 2 (line 314) |
| It is not clear which variables are controlled in the multivariate model. This makes it very difficult to infer the effects of perceived social norms and beliefs on contraceptive use. A more precise way to handle this would to running a separate model with the main independent variables without controls and another with the controls. | Taking your comment in to account, we re-run a separate regression model with the main independent variables without controls and then run the final model (model 2) to control for all variables and showed p-value <=.25 in bivariate analyses. It is described in data analysis section and table 2 of the revised manuscript. | Variable and measurement section, line 202-205; data management and analysis section, 263-267, Table 2 |
| Review how the logistic regression results are reported. There seems to a confusion between the outcome and independent variables in the way the odds are reported. It is, therefore, very difficult to understand the magnitude and the direction of the effect of the independent factors on the outcome. | We have accepted the suggestions and it is described now in the conventional way of reporting. | Abstract, line 46-53  Result, line 302-310 |
| **Reviewer #2:** |  |  |
| In the result section mean and standard deviations should be reported rather than SE. | Thank you for showing the error! ‘SE’ was typing error which was to mean SD (standard deviation), now it is corrected in the revised manuscript. | Result  line 278, 280 |
| Discussion: there is low contraceptive utilization in your study area even compared to the surrounding areas. You tried to tell us the presence of persistent socio-cultural norms that encourages large family size and then non-use of contraceptives methods at young age in rural setting. But the utilization is still lower than other rural areas. I suggest to find out more on the possible factors that contributed low utilization in your study area. | We have tried to indicate more clues that could contributed to contraceptive utilization in the revised manuscript. | Discussion,  line 333-338 |
| **Reviewer #3:** |  |  |
| 1. Introduction: In the final statement of the introduction, in which you describe the aim of the study, it would be beneficial to describe this in more detail, so that you are very clearly stating the aim and the scope of the paper. Stating that the aim is to assess contraceptive behavior is perhaps too broad. | Thank you for important comment and suggestions! We have addressed your concern in the revised manuscript in a more clear way it would help to get the idea. | Introduction,  The last 2 paragraph |
| 2. Study setting: The description of the study site is very useful. It would be helpful to list the statistics you listed for the site for Ethiopia in general, so we can compare it to the average for the country. This will help to put your findings in context. | Good reminder! We have included nationally available information in the revised manuscript | Study setting  Line 122-127 |
| 3. Methods and materials: It would be helpful to clarify how the sample was actually chosen. This section on sampling would benefit from the actual equation used with the inputs provided (contraceptive utilization, precision, design effect, and non-response). Why were all women included if the sample size was far exceeded? | Thank for the point! We have addressed your concern is in the revised manuscript. | Study population,  line 131-140 |
| Were women contacted in their homes (was this a household survey?) | Thank you for raising the issue. Women were contacted in their homes. Now we clarified it in the revised manuscript. | Data collection,  Line 149-151 |
| Additionally, since half of the sample had no education, it may be useful to clarify how written informed consent was obtained for those who may not be able to read/write. | We appreciate your concern! The issue is clarified in the revised manuscript. | Ethical consideration,  Line 157-159 |
| 4. Variables and measurements: It would be very helpful to understand whether these injunctive norms and descriptive norms fit into a conceptual framework of some sort. How are these norms theorized to affect behavior? It would be very helpful to have a description of these norms/their expected effect on behavior in the introduction section of this paper, to help frame the research you conducted to the audience. Please also specify what all of the response options are for each of the norm and myths and misconception variables. | We have corrected your concern in introduction accordingly.  The response option of the items used for measuring the main independent variables is indicated in S1 Table and in the ‘variables and measurement’ section | Introduction,  Line 90-107  S1 Table,  Variables and measurement,  Line 177, 178, 195 |
| Did you hypothesize any sort of relationship between your two main predictor variables? It would be helpful to explain how you think the norms as well as the belief in myths/misconceptions might be related, in the introduction. Did you predict that one would be more important than the other, or that while they might be independently associated with the outcome, one would no longer be significant in multivariate analyses? | The relationship between the two variables was evaluated using correlation analysis, and it didn’t show any significant correlation. So, we included both variables in the final multivariate analysis. Hence, each variable associated with contraception independently as shown in the manuscript. |  |
| There are a lot of scale variables included, especially for variables that are not main predictors for the model. You may consider reassessing whether all of these are needed.  Additionally, if you decide to keep them all, please elaborate on the items included in the contraceptive knowledge scale. | **Adjusting for all possible variables is helpful to indicate the independent influence of the main independent variables with the outcome variable. It also adds scientific information regarding other possible factors that could influence young women's contraception.**  **Considering your suggestion, all the items included in the contraceptive knowledge scale are included in the revised manuscript as supporting information.** | **S2 Table** |
| 5. Data analysis: Is table 2 necessary for helping to understand the paper? I didn’t understand the purpose of this analysis from the description in the methods, perhaps because you had already described how the scales were calculated in the previous section of the methods. The Cruide ORs Table 2 may be too much information, and could be considered for an appendix if you decide it is needed. If it is needed, please elaborate on its purpose more in this section of the methods. I also don’t think you need to refer to the table numbers in the methods section. | We have revised table 2 and included as supporting information in the revised manuscript. | S1 Table |
| The sentences where you describe that the scale variables were all included as continuous variables in the model is confusing because you describe how these variables were developed/coded in the variables and measurement section. I think you could put these sentences in the variables section instead.  The data analysis section should also clearly state that multivariate logistic regression was conducted, I don’t think this statement was included. | This is good suggestion and we have corrected it accordingly in the revised manuscript |  |
| 6. Results: The frequencies for age in Table 1 do not add up to 100. If there are missing data, please clarify this.  On the other hand, the frequencies add up to more than your total sample size. Please clarify. | We apologize for the error. It was a typing error, as it can be seen in the frequency column. We typed 39.5 to type 59.5. Now, it is corrected.  As indicated in the result, out of 3102 young women identified from the database, 3039 responded to the question about the outcome making 2.1% non-response rate. Some of those non-respondent were discontinued the interview after interviewed the questions on socio-demographic variables and before responding to the question on contraceptive utilization. Hence, the number of participants that responded to socio-demographic questions were above the sample size, now we have corrected by limiting to the number of participants who responded to the outcome variable. | Result ,  Table 1, line 286  Result,  Line 287-288 |
| For the duration of continuous utilization, is that among current users only? | Good point. It was reported for those currently using contraceptive. We have clarified it in the revised manuscript. | Result,  Line 294-295 |
| I don’t understand the purpose of the Cruide ORs from Table 2 in the body of the paper, perhaps it could be included in an appendix. | We have revised and submitted it as supporting information in line with your recommendation. | S1 Table |
| 7. Results: The text describing Table 3 needs to be heavily revised. The odds ratios are not described in the correct way – the odds are of current contraceptive use, not of the background characteristics. Please revise here and in the abstract and discussion as well. | Taking your comment into account, we have revised each section in the revised manuscript. | Abstract, line 46-53  Result, line 302-310  Discussion , 343-346, 373-375 |
| Please also continue to be very clear about the outcome and include “current” in contraceptive use. | Good reminder! We have included it accordingly in the revised manuscript. | Abstract, line 45  Discussion, 343, 374  Result, line 294, 295… |
| It would also be helpful to show the reference groups for all variables, even those with 2 categories. Then you could also add the Frequency (%) of contraception for each variable. | Thanks. We agree and added reference groups for all categorical variables. For continuous variables, however, we have added mean of contraceptive users and non-users. | Table 2 |
| I would also put the main predictor variables at either the beginning or the end of the table so they stand out. | That is a good suggestion and we have corrected it accordingly. | Table 2 |
| 8. I think the discussion clearly explores a lot of the main findings from the results. In the paragraph on descriptive norms, in the second sentence, it’s unclear whether the authors are arguing that it’s a misperception that most young women aren’t using contraception, when this study showed only 14% were using. Please elaborate on this possible reason women aren’t using. As I mentioned above, it would be helpful to elaborate on the relationships between these two norms as well as the relationship between norms and believing in myths. Please also spend a bit more time elaborating on suggested interventions and future research, perhaps among positive deviants. | Thank you! You are right, the sentence was not clear enough to understand. Now, we have clarified the sentence and also elaborate the possible reasons for not using as well as about the suggested interventions and further study pointed in the paragraph. | Discussion,  line 360-369; 381-383; 397-404 |
| I’m not sure I understand this sentence in the limitations: “Additionally, it could be argued that the positive association between contraceptive use and subjective norm may be due to contraceptives users may more likely to perceive others’ approval than who were not using.” Please elaborate. | Good point. We have more clarified the message of the sentence in the revised document | Discussion,  Last paragraph 395-401 |
| Was there a minimum age for inclusion in this study? | No minimum age limit, rather defined as all married young women aged less than or equal to 24 years |  |
| Figure 1 is very hard to read. | We have revised the figure the way it would be easy to understand. | Fig. 1 |
| I think this study would strongly benefit from a copy-edit for clarity, grammar, and minor issues with formatting in the text (this, along with the description of the ORs as mentioned above is the reason I selected No for Question) | Good suggestion and repeatedly edited for grammar and clarity |  |
| PLOS One author guidelines state that contacting the corresponding author for data is not sufficient to meet their data availability guidelines. | Thank you for your reminder. The anonymized data set is uploaded |  |
| **Academic Editor** |  |  |
| 1. Please ensure that your manuscript meets PLOS ONE's style requirements, including those for file naming | We have checked the revised manuscript for the PLOS ONE's style requirements |  |
| 2. Please include additional information regarding the survey or questionnaire used in the study and ensure that you have provided sufficient details that others could replicate the analyses. | We have included all the necessary information regarding the survey or questionnaire used in the study in the manuscript. |  |
| 3. You indicated that you had ethical approval for your study.  In your Methods section, please ensure you have also stated whether you obtained consent from parents or guardians of the minors included in the study or whether the research ethics committee or IRB specifically waived the need for their consent. | Good point! Now we have included in the revised manuscript under new sub-heading ‘Ethical consideration’. | Methods and material section.  Line 154, 157-161 |
| 4. During your revisions, please note that a simple title correction is required to ensure there are no errors of grammar: "Low contraceptive utilization among young married women is associated with perceived social norms and believing in contraceptive myths in rural Ethiopia". | Thank you for your important reminder! We have corrected it. | Title |
| 5. We note that you have indicated that data from this study are available upon request. PLOS only allows data to be available upon request if there are legal or ethical restrictions on sharing data publicly.  If there are no restrictions, please upload the minimal anonymized data set necessary to replicate your study findings as either Supporting Information files or to a stable, public repository and provide us with the relevant URLs, DOIs, or accession numbers | The necessary anonymized data set is uploaded as Supporting information |  |
