# Supplementary material for: Low contraceptive utilization among young married women is associated with perceived social norms and belief in contraceptive myths in rural Ethiopia
Source: PLoS One. 2021 Feb 22;16(2):e0247484. doi: 10.1371/journal.pone.0247484 (PMC7899365; doi:10.1371/journal.pone.0247484)
Supplement: Attachment — Submitted filename: Response to Reviewers.docx [file pone.0247484.s006.docx]

October 06, 2020

José Antonio Ortega, Ph.D.

Academic Editor-PLOS ONE

Re: Manuscript Sub ID No. PONE-D-20-05351

Please find response to each point raised by the academic editor and reviewers, and a revised version of our manuscript “Low contraceptive use, perceived social norms and belief in contraceptive myths among young married women in Eastern Ethiopia”, which we would like to resubmit for publication as an original article in PLOS ONE.

Your comments and those of the reviewers were highly insightful and enabled us to greatly improve the quality of our manuscript. Our point-by-point response to each of the point raised is attached with this letter. We hope that the revisions in the manuscript and our accompanying responses will be sufficient to make our manuscript suitable for publication in the PLOS ONE.

We shall look forward to hearing from you at your earliest convenience.

Yours sincerely,

**Title:** **Response to Reviewers comment of the Manuscript entitled “Low contraceptive use, perceived social norms and belief in contraceptive myths among young married women in Eastern Ethiopia”**

| **S.no** | **Reviewer comment/Recommendation** | **Authors Response** | **Section where correction made** |
| --- | --- | --- | --- |
| 1 | One of the characteristics that set apart this analysis from others is the focus on young AND married women. There should be some reference on pressure to procreate once married (i.e: <https://doi.org/10.20897/femenc/5918>, <https://doi.org/10.1017/S0021932000003552>). When this is understood, the low contraceptive prevalence of this group is understood better: they want to have children, particularly their first child. Probably the “policy” recommendation would not be so much “use contraceptives after getting married” but “marry later” given this cultural association. This should be highlighted to understand the context of such low contraceptive use as commented by reviewer | Thank you for this good point! We have addressed it the revised manuscript based on your suggestion. | Line 337-340 and 347-348, 388 |
| 2 | Also, in that respect, it would help to give comparable statistics on contraceptive prevalence for a similar group in Ethiopia from DHS. [I have seen you do it in the discussion comparing with DHS2016 in l. 329-330, I still give the data I looked for MiniDHS2019] For instance, it can be computed from the MiniDHS2019 (<https://dhsprogram.com/pubs/pdf/PR120/PR120.pdf>). It shows 36.5 prevalence for 474 currently married women 15-19 and 52.2 prevalence for 951 married women 20-24. Based on this a proportion for married women 15-24 can be estimated and provided as a reference. Indeed CP is very low in this region. While DHS does not provide a figure for Kersa, CP is not low in Oromia (40.7 compared to 41.4 nationally). However, neighbouring Ethiopia Somali shows only has 3.4 CP. Is there some clear a priori reason for this low uptake? | Good point. We have taken the comment into account and tried to address it in the discussion section of the revised manuscript. | Line 333-337 and 340-342 |
| 3 | Differences could also arise due to a different protocol in asking about contraceptive use. This should be acknowledged somewhere as a limitation since the DHS protocol is the standard for measuring CP internationally. Difference might lie in that DHS, before asking about use, asks about knowledge of each method. | We understand your concern. However, the data collection approach had no significant difference with DHS concerning contraceptive knowledge, and utilization other than our data collection tool was even more detailed and specific. Hence, the difference in data collection protocol is unlikely to be the cause of the difference in the level of contraceptive utilization. |  |
| 4 | In contrast, line 333-334 is too vague: “the persistent socio-cultural norms that encourages large family size and then non-use of contraceptives methods at young age in rural setting of developing country”. There are many rural setting in developing countries where it is not the case. Also phrasing in singular form is not correct. Maybe “the persistent socio-cultural norms that encourage large family size and then non-use of contraceptive methods at young ages in this rural setting” | Thank you. Revise it the way more clear in the revised manuscript. | Line 340-345 |
| 5 | There is no clear information on when the survey was carried out. That should be made clear. | Sorry for failing to indicate in an easily visible way. It was stated in the last line of the paragraph about ‘Study setting’. Now we have added the word “period” to the subtitle "study setting" and change to “Study setting and Period” to make it visible. | Line 114 and 126-127 |
| 6 | I also see missing data in the dataset. I guess most correspond to women that are not married. How handling of missing data was carried out should be mentioned in the methods section. | Thank you for raising the issue! Data were collected only from young married women. Hence, the missed data was not that of not married women. Now we have included missing data management within the method section. | Line  261-263 |
| 7 | I understand your argument of multicollinearity of age and parity, also raised by referee 1, but for the reason given above (pressure to become mother), it would be good to include at least a variable distinguishing ever-mothers. One would expect the variables of age lose importance after including it, but it is ok. Also much lower CP for non-mothers would be expected. Please include the variable in the multivariate analysis since non-mothers are less likely to use contraception. Note that l.205 says you are controlling by parity which seems not to be the case (after the addition of the variable, it will). | We appreciate your concern. As we have tried to address in the previous comment, there is a strong relationship between age and parity to include both variables together in the multivariate analysis. However, taking into account the importance of your comment, we have included it in the result section which we believe it could help to understand the low prevalence of contraception among never mother young women. | Line  300-301 |
| 8 | The title has been changed but it still does not read correctly. “Believing” should be “belief”, and it still reads too long. I would suggest “Low contraceptive use, perceived social norms and belief in contraceptive myths among young married women in Eastern Ethiopia”. | Thank you! We have revised it based on your recommendation. | Title |
| 9 | There is no need to put all results in the abstract, just the main ones, in lines 45-53. | Good point. Now taken into account your comment, we revised and reduced to main findings. | Line 44-50 |
| 10 | The text would still requires copy-editing. Some suggestions in that respect, focusing in the abstract, but there is a need to check everything, particularly the discussion. | Thank you for the detailed comments and recommendations which tremendously help us to improve the manuscript. In addition to editing the whole section, each point you have raised is corrected in the revised manuscript. |  |
|  | - l. 39-40: “aged 14-24” instead of in the age-group 14-24. |  | Line 38 |
|  | - L. 45: Remove “the” in the “the perceived social approval” |  | Line 44 |
|  | - L. 56: Remove “the” in “the sociocultural…”. |  | Line 54 |
|  | - L. 57: Replace “are necessary” which does not stem from the analysis, and “to improve”, which assumes a value judgement regarding contraceptive use, with “could increase” or something similar. |  | Line 55 |
|  | - Avoid “unmet needs” (l. 73), use “unmet need”. |  | Line 74 |
|  | - L. 336: suppress “norm” |  | Line 344 |
|  | - L. 339-40: Rephrase. “Despite” does not make sense. Also “imperative” is too strong |  | Line 338-340 |
|  | - L. 364 should be “mothers |  | Line 344 |
|  | - L. 366: suppress “be” |  | We made detailed editing to this paragraph. |
|  | - L. 367-68 do not make sense. Rephrase |  | Revised 368-376 |
|  | - L. 375: Again the problem with “belief” (noun) and “believe” (verb) but the other way round. It should be “believe in” instead of “belief the” |  | It is corrected throughout the manuscript where necessary. |
|  | - L. 379: Again rephrase “developing countries”. This example is not representative. Just suppress “in developing countries”. Also “which even accompanied by” is wrong. In general this paragraph needs copy-editing |  | Line 382-385 |
|  | - L- 388 Also check all this page for grammar. Example: “This tended to resulted” maybe “result”. You can use “Grammarly.com” or a similar tool that will indicate all these problems. | Thank you! The suggestion is well taken and utilized | Lin3 392 and other sections |
|  | L. 410: Socio-cultural, not social-cultural. Also commas missing before mainly and after myths |  | Line 444 |
