# Supplementary material for: Low contraceptive utilization among young married women is associated with perceived social norms and belief in contraceptive myths in rural Ethiopia
Source: PLoS One. 2021 Feb 22;16(2):e0247484. doi: 10.1371/journal.pone.0247484 (PMC7899365; doi:10.1371/journal.pone.0247484)
Supplement: Attachment — Submitted filename: Response to Reviewers.docx [file pone.0247484.s007.docx]

October 30, 2020

José Antonio Ortega, Ph.D.

Academic Editor-PLOS ONE

Re: Manuscript ID No. PONE-D-20-05351R2

Please find response to each point raised by the academic editor and/or reviewers, and a revised version of our manuscript “Low contraceptive use, perceived social norms and belief in contraceptive myths among young married women in Eastern Ethiopia”, which we would like to resubmit for publication as an original article in PLOS ONE.

Your comments and those of the reviewers were highly insightful and enabled us to greatly improve the quality of our manuscript. Our point-by-point response to each of the point raised is attached with this letter. We hope that the revisions in the manuscript and our accompanying responses will be sufficient to make our manuscript suitable for publication in the PLOS ONE.

We shall look forward to hearing from you at your earliest convenience.

Yours sincerely,

**Title:** **Response to Reviewers comment of the Manuscript entitled “Low contraceptive use, perceived social norms and belief in contraceptive myths among young married women in Eastern Ethiopia”**

| **S.no** | **Reviewer comment/Recommendation** | **Authors Response** | **Section where correction made** |
| --- | --- | --- | --- |
| 1 | 3- Possible limitation, differences with DHS protocol.  You argue that this is not an issue, but I cannot judge this based on the information I have. I am concerned, for instance, about probing questions regarding knowledge that can make a difference (and are known to make a difference). Please provide a copy of the questionnaire so that this can be assessed. If the protocol is exactly the same, please make it clear in the paper If it is different, acknowledge that as a possible limitation and describe in what ways it is different. | We understand your concern and describe the similarity of our questions with the DHS questionnaire in the revised manuscript. A copy of the DHS questionnaire is found on the following website: <http://dhsprogram.com/pubs/pdf/FR328/FR328.pdf> .  The questionnaire specific to contraception is indicated on ‘**APPENDIX E’**, under the title ‘**SECTION 3. CONTRACEPTION**’ and page 402 or pdf page 435 | Line 143-144 and 347-348, 388 |
| 2 | I insist in the model with an ever-mother covariate. You could include both models for comparison, but I think it is even more important after showing the strong bivariate link, As I said there are important reasons to include it: married youth with no children are very likely to desire getting pregnant and, therefore, not to use contraception. | We appreciate your concern and addressed it by including both models (age and ever-mother status) based on your suggestion. | Line  318-323 |
