# Supplementary material for: Low contraceptive utilization among young married women is associated with perceived social norms and belief in contraceptive myths in rural Ethiopia
Source: PLoS One. 2021 Feb 22;16(2):e0247484. doi: 10.1371/journal.pone.0247484 (PMC7899365; doi:10.1371/journal.pone.0247484)
Supplement: Attachment — Submitted filename: Response to Reviewers.docx [file pone.0247484.s008.docx]

November 18, 2020

José Antonio Ortega, Ph.D.

Academic Editor-PLOS ONE

Re: Manuscript ID No. PONE-D-20-05351R2

Please find response to each point raised by the academic editor and/or reviewers, and a revised version of our manuscript “Low contraceptive utilization among young married women is associated with perceived social norms and believing in contraceptive myths in rural Ethiopia”, which we would like to resubmit for publication as an original article in PLOS ONE.

Your comments and those of the reviewers were highly insightful and enabled us to greatly improve the quality of our manuscript. Our point-by-point response to each of the point raised is attached with this letter. We hope that the revisions in the manuscript and our accompanying responses will be sufficient to make our manuscript suitable for publication in the PLOS ONE.

We shall look forward to hearing from you at your earliest convenience.

Yours sincerely,

**Title:** **Response to Reviewers comment of the Manuscript entitled “Low contraceptive utilization among young married women is associated with perceived social norms and believing in contraceptive myths in rural Ethiopia”**

| **S.no** | **Reviewer comment/Recommendation** | **Authors Response** | **Section where correction made** |
| --- | --- | --- | --- |
| 1 | - ISSUE 1: 3- Possible limitation, differences with DHS protocol.  You argue that this is not an issue, but I cannot judge this based on the information I have. I am concerned, for instance, about probing questions regarding knowledge that can make a difference (and are known to make a difference). Please provide a copy of the questionnaire so that this can be assessed. If the protocol is exactly the same, please make it clear in the paper If it is different, acknowledge that as a possible limitation and describe in what ways it is different.  RESPONSE: You are giving me a link to the DHS questionnaire. I know that one. What I was asking was to provide a copy of YOUR questionnaire. I am still unable to assess what I was mentioning. And, as I said, it is not a question of similarity. If it is not identical in the protocol (example: probing with knowledge of methods,) you have to acknowledge that as a possible limitation and describe in what ways it is different. That is still missing. | We appreciate your concern and thank you for your effort to improve the quality of our manuscript. We understand the possibility of some differences in data collection protocol with DHS as you have suggested but couldn’t identify differences specific to asking contraceptive knowledge and use to describe more in the manuscript. Thus, we have provided a copy of part of the questions used to ask contraceptive knowledge and use with the revised manuscript. |  |
| 2 | ISSUE 2: I insist in the model with an ever-mother covariate. You could include both models for comparison, but I think it is even more important after showing the strong bivariate link, As I said there are important reasons to include it: married youth with no children are very likely to desire getting pregnant and, therefore, not to use contraception.  RESPONSE: We appreciate your concern and addressed it by including both models (age and ever-mother status) based on your suggestion.  I welcome the addition of the model. You must see how important the coefficient is on ever-mother. But, still, the model is not commented in the text. You should. Please go back to my original comments on the reason for my insistence with including the variable:  I understand your argument of multicollinearity of age and parity, also raised by referee 1, but for the reason given above (pressure to become mother), it would be good to include at least a variable distinguishing ever-mothers. One would expect the variables of age lose importance after including it, but it is ok. Also much lower CP for non-mothers would be expected. Please include the variable in the multivariate analysis since non-mothers are less likely to use contraception. Note that l.205 says you are controlling by parity which seems not to be the case (after the addition of the variable, it will).  Please comment on this in the main text. Please also change the names of the models that do not read well. You can call them model 2a and model 2b or models 2 and 3 or something like that. Please also add some measure of model fit for models 1, 2a and 2b that could be used to choose among them, such as AIC, BIC, Nagelkerke pseudo R-squared, etc. Make sure that the results reported throughout the text refer to the best model and make it clear in the text. | We hope your concerns and comments are well addressed in the revised manuscript staring from the abstract section. | Line  49-50,  205-206,  274-277,  318-319,  328-333,  343-352 |
