# Supplementary material for: Low contraceptive utilization among young married women is associated with perceived social norms and belief in contraceptive myths in rural Ethiopia
Source: PLoS One. 2021 Feb 22;16(2):e0247484. doi: 10.1371/journal.pone.0247484 (PMC7899365; doi:10.1371/journal.pone.0247484)
Supplement: Attachment — Submitted filename: Response to Reviewers.docx [file pone.0247484.s009.docx]

December 25, 2020

José Antonio Ortega, Ph.D.

Academic Editor-PLOS ONE

Re: Manuscript ID No. PONE-D-20-05351R2

Please find response to each point raised by the academic editor and/or reviewers, and a revised version of our manuscript “Low contraceptive utilization among young married women is associated with perceived social norms and belief in contraceptive myths in rural Ethiopia”, which we would like to resubmit for publication as an original article in PLOS ONE.

Your comments and those of the reviewers were highly insightful and enabled us to greatly improve the quality of our manuscript. Our point-by-point response to each of the point raised is attached with this letter. We hope that the revisions in the manuscript and our accompanying responses will be sufficient to make our manuscript suitable for publication in the PLOS ONE.

We shall look forward to hearing from you at your earliest convenience.

Yours sincerely,

**Title:** **Response to Reviewers comment of the Manuscript entitled “Low contraceptive utilization among young married women is associated with perceived social norms and belief in contraceptive myths in rural Ethiopia”**

| **S.no** | **Reviewer comment/Recommendation** | **Authors Response** | **Section where correction made** |
| --- | --- | --- | --- |
| 1 | Regarding the questionnaire, you convinced me that you were following the same DHS protocol.  I suggest changing the current sentence "We followed the DHS questionnaire protocol in adapting the questions used to assess contraceptive knowledge and utilization" with something more to the point "We followed the DHS questionnaire protocol for questions on contraceptive knowledge and utilization" | That sounds a good suggestion and we have corrected accordingly. | Line 143-144 |
| 2 | I think my instructions were clear "Please also add some measure of model fit for models 1, 2a and 2b that could be used to choose among them, such as AIC, BIC, Nagelkerke pseudo Rsquared, etc. Make sure that the results reported throughout the text refer to the best model and make it clear in the text.”  What you have done is to include a sentence that reads confusing "We used Akaike’s information criterion (AIC), Bayesian information criterion (BIC), and Pseudo R2 in building the regression models. The model with low AIC and BIC, and high Pseudo R2 scores were considered as the final model. Additionally, the model fitness tested using the Hosmer–Lemeshow goodness-of-fit tests". There is no other mention of AIC, BIC or Pseudo R2 in the text. What is going on?  I will be more clear:  1. Choose ONE model selection criterium. I'd suggest AIC.  2. Report in table 2 the AICs for the different models. In particular look at what model is best according to the criterium: model 2a or model 2b, and make notice of it in the main text. | Thank you! We have revised it based on your recommendation. | Line 267-278  Line 323-324 (end of table 2) |
| 3 | Make sure that all references in the text and abstract to results from the models refer to the best model from step 2. | Addressed in the revised manuscript. | Throughout the manuscript |
| 4 | COMEBACK ISSUE: The title changed again. It is OK except for "believing" that should be BELIEF. | We have changed it in the revised manuscript. | Title |
| 5 | NEW ISSUE: Since you are highlighting in the paper the connection with the variables on social pressure and contraceptive myths, I see that in the "limitations" paragraph (399-416) there is no mention regarding possible alternative interpretations of the contraceptive myths variable which are clearly there in an observational setting such as post-hoc rationalization. In particular you should add something along these lines "Regarding the impact of belief in contraceptive myths, it cannot be ruled out that the relationship is not causal but due to post-hoc rationalization of a refusal to use contraception". | Yes, we agree, that is now taken into account and corrected in the revised manuscript. | Line 409-412 |
