# Supplementary material for: Low contraceptive utilization among young married women is associated with perceived social norms and belief in contraceptive myths in rural Ethiopia
Source: PLoS One. 2021 Feb 22;16(2):e0247484. doi: 10.1371/journal.pone.0247484 (PMC7899365; doi:10.1371/journal.pone.0247484)
Supplement: Attachment — Submitted filename: Response to Reviewers.docx [file pone.0247484.s010.docx]

January 20, 2021

José Antonio Ortega, Ph.D.

Academic Editor-PLOS ONE

Re: Manuscript ID No. PONE-D-20-05351R2

Please find response to each point raised by the academic editor and/or reviewers, and a revised version of our manuscript “Low contraceptive utilization among young married women is associated with perceived social norms and belief in contraceptive myths in rural Ethiopia”, which we would like to resubmit for publication as an original article in PLOS ONE.

Your comments and those of the reviewers were highly insightful and enabled us to greatly improve the quality of our manuscript. Our point-by-point response to each of the point raised is attached with this letter. We hope that the revisions in the manuscript and our accompanying responses will be sufficient to make our manuscript suitable for publication in the PLOS ONE.

We shall look forward to hearing from you at your earliest convenience.

Yours sincerely,

**Title:** **Response to Reviewers comment of the Manuscript entitled “Low contraceptive utilization among young married women is associated with perceived social norms and belief in contraceptive myths in rural Ethiopia”**

| **Reviewer/Editor comments** | **Authors Response** |
| --- | --- |
| A first problem of factual incorrection that needs to be rewritten is this:  72 Previous studies have reported the highest levels of unmet need, and the lowest and interrupted  73 contraceptive utilization among young married women in developing countries [8].  The cited study does not say what is being said here. It is not a comparative study, it only studies unmet need of young women. In addition, it is not true that young married women have the highest unmet need levels. For instance, <https://www.demographic-research.org/volumes/vol38/45/38-45.pdf> show that sexually active unmarried young-women have higher levels of demand and unmet need in most DHS surveys than their married counterparts. If we look at unmet need by age in Ethiopia, for instance DHS 2016, in table 7.10.1 we find that unmet need is highest for married women 35-39, and in table 7.10.2 the same applies to all sexually active women. Table 7.10.2 indicates that 15-19 and 20-24 actually have the lowest levels of unmet need.  Regarding editing, I provide a detailed assessment of problems. In particular the section on variables and measurement is written very poorly. Throughout the text there remain problems of grammatically incorrect sentences. I give you a detailed list of problematic sentences and proposed solutions, but the final text must be checked throughout afterwards. I also insert a couple of problems that are not only grammatical.  85 associated with women’s contraceptive utilization [15-18]. Studies have also been reported the  85 associated with women’s contraceptive utilization [15-18]. Studies have also reported the  89 Recently, literature increasingly recognizing the strong influence of perceived social norms and  89 Recently, literature increasingly recognizes the strong influence of perceived social norms and  91 perceived social norm is commonly shared beliefs about most people in their social networks’ (reference group) approval of contraceptive practice 92 (injunctive norm), and believe that most  93 people in their network contraceptive behavior (descriptive norm)  91 perceived social norm is commonly shared beliefs regarding people in their social networks’ (reference group) approving of contraceptive practice 92 (injunctive norm), or believing that most  93 people in their network contraceptive behavior (descriptive norm)  94 parents, and husbands' approval influences married young women’s contraceptive utilization  94 parents, and husbands' approval influences young married women’s contraceptive utilization  96 methods [22, 27, 28]. The perception of contraceptives behavior of Friends  96 methods [22, 27, 28]. The perception of contraceptive behavior of friends  98 friends using contraceptives  98 friends are using contraceptives  Moreover,  99 studies have been shown the influence of belief in myths and misperception or beliefs of rumors  100 not supported by evidence about contraceptive methods on young married women’s contraceptive  101 use [23, 30-35].133 questions used to measure the exposure variables and a large number of variables assessed like  Moreover,  99 studies have been shown the influence on young married women’s contraceptive use of belief in myths and misperception or beliefs in rumors  100 not supported by evidence regarding contraceptive methods  [23, 30-35].    In a community-based survey in which multiple Likert scale  133 questions used to measure the exposure variables and a large number of variables assessed like  134 this study, having the largest possible sample size    In a community-based survey such as this one, where multiple Likert scale  133 questions are used to measure the exposure variables and there are a large number of covariates, having the largest possible sample size …    135 error within  135 error even within  147 women who were residing in a similar setting outside of the study area. The female data collectors  147 women who were residing in a similar setting outside of the study area. Female data collectors  150 participants and who consented  150 participants who consented  164 The outcome variable was the current contraceptive utilization.  164 The outcome variable was current contraceptive utilization.  165 their spouse were currently using any contraceptive method to prevent or delay pregnancy. Those  166 responded ‘yes’  165 their spouse were currently using any contraceptive method to prevent or delay pregnancy. Those  166 who responded ‘yes’  178 in-law believe I should use contraceptive”.  178 in-law believe I should use contraceptives”.  179 strongly agree (5) and the index score was constructed by summing the items score and dividing  180 the total scores for the total number of items 179 strongly agree (5) and the index score was constructed by dividing  180 the total score by the total number of items  180 the total scores for the total number of items (S2 Table). The possible score range from 1-5 with  180 the total scores for the total number of items (S2 Table). The possible score ranges from 1-5 with  181 a higher score indicates that participants perceive their referents are more approve of their  181 a higher score indicating that participants perceive their referents approve more their  184 practice(descriptive norm) was assessed by single item  184 practice(descriptive norm) was assessed by a single item  186 using contraceptive method  186 using contraceptive methods  187 score indicates the stronger the likelihood that respondents thought their peers were using the  188 contraceptive method.  187 score indicates the stronger the likelihood that respondents thought their peers were using  188 contraceptive methods.  192 The statement include  192 The statements include  197 by summing the item scores and divided the total score for the number of ítems  197 by summing the item scores and dividing the total score by the number of items  198 scores range from 1-5,  198 score is in the range from 1 to 5,  203 To control for possible confounding covariates including contraceptive self‐ efficacy, knowledge**,**  204 inter**-**spousal communication, household decision-making autonomy, recent exposure to FP  205 information and socio-demographic variables such age, educational status, parity (ever206  mother/never-mother), desired number of children and wealth status were included the  207 multivariate analysis model.  203 The multivariate analysis controls for possible confounding including as covariates contraceptive self‐ efficacy, knowledge**,**  204 inter**-**spousal communication, household decision-making autonomy, recent exposure to FP  205 information and socio-demographic variables such age, educational status, parity (ever206  mother/never-mother), desired number of children and wealth status.    209 Contraceptive self‐ efficacy was measured by four items asked  209 Contraceptive self‐ efficacy was measured by four items asking  210 how confident or sure they could use contraceptive and insist husband’s  210 how confident or sure they were they could use contraceptive and insist the husband  211 items include how sure that the woman : could tell her your husband that wanted use of family  212 planning, … could use family planning, … could discuss topic of family planning with husband,  213 and … could use family planning even if her husband did not want. The items responses  211 items include how sure was the woman that : she could tell her husband that she wanted to use family  212 planning, … she could use family planning, … she could discuss topics of family planning with her husband,  213 and … she could use family planning even if her husband did not want. The item responses  216 by the number of item[45]. The scale score range from 1-5  216 by the number of items[45]. The scale score ranges from 1-5  217 level of confidence to use contraceptive methods. The scale had good reliability scale (Cronbach's  217 level of confidence to use contraceptive methods. The scale had good reliability (Cronbach's  220 Spousal communication was measured using a 4-items to ask the respondent how often woman  220 Spousal communication was measured using 4-items asking the respondent how often the woman  221 may discuss things with her husband [45]. Each woman was asked how often she discuss with  221 may discuss things with her husband [45]. Each woman was asked how often she discusses with  223 have, and whether to use contraceptive methods. The item response option ranges  223 have, and whether to use contraceptive methods. The item response options range  230 a range of key household decisions that affect her life and measured using the 10-items scale  230 a range of key household decisions affecting her life and measured using the 10-items scale  237 Contraceptive knowledge was another covariate assessed using 15 items adapted from literature  238 and emphasized on knowledge of specific contraceptive methods  237 Contraceptive knowledge was another covariate assessed using 15 items adapted from the literature  238 and emphasizing knowledge of specific contraceptive methods  240 obtained by summing all items. The score range from  240 obtained by summing all items. The score ranges from  245 whether they were told about FP during their visit to health facility, to Health Extension Worker  246 (HEW) or others Health care Workers (HCWs) who visited their home  245 whether they were told about FP during their visit to a health facility, or by a Health Extension Worker  246 (HEW) or other Health care Workers (HCWs) who visited their home  251 Wealth index was computed by employing principal component analysis (PCA) using items  252 assessed household facilities and possessions.  251 Wealth index was computed by employing principal component analysis (PCA) based on items  252 assessing household facilities and possessions.  254 each component. The score of the first component or factor which comprised of several heavily  255 loaded variables and accounted for the largest variation in the data was categorized into quintiles  256 where each individual fall into    254 each component. The score of the first component or factor comprising several heavily  255 loaded variables and accounting for the largest variation in the data was categorized into quintiles  256 where each individual falls into    257 Additionally, socio-demographic characteristics including current age, age of marriage  258 educational status, residence of living (rural and urban), alive and ideal number of children were  259 included in the analysis.    257 Additionally, socio-demographic characteristics included in the analysis are current age, age at marriage,  258 educational status, place of residence (rural and urban), alive and ideal number of children    272 to the multicollinearity  272 to multicollinearity    275 models. The AIC values were compared in successive models, and the lowest value was considered  276 the best-fit model. The value of AIC showed subsequent reduction throughout the model building  277 which indicates each model improvement over the previous, illustrating the goodness of fit of the  278 final regression model (Table 2).    275 models. The model with the lowest AIC value is model 2b, including parity and excluding age.  (Table 2).  NOTE. They are non-nested models. It is not the subsequent reduction: it is one or the other.    296 **Abbreviations:** ANC, antenatal car;  296 **Abbreviations:** ANC, antenatal care    300 24.6%). Among the study participants, 369 (14.2%, 95% CI: 12.8%-15.5%) were currently using  300 24.6%). Among the study participants, 369 (14.2%, 95% CI: 12.8%-15.5%) were currently using a    306 currently using contraceptive methods (p-value <0.001). More than two-third  306 currently using contraceptive methods (p-value <0.001). More than two-thirds    308 **Fig 1. This is current contraceptive utilization**  308 **Fig 1. Current contraceptive utilization**;    315 utilization **(**AOR= 0.60; 95% CI: 0.49-0.73). Moreover, odd of contraceptive utilization was  315 utilization **(**AOR= 0.60; 95% CI: 0.49-0.73). Moreover, odds of contraceptive utilization were    318 (AOR=9.68; 95% CI: 4.47-20.90), and attended secondary education  318 (AOR=9.68; 95% CI: 4.47-20.90), and who attended secondary education    332 associated young women contraceptive utilization. Moreover, the more young women belief in in  333 contraceptive myths was negatively associated with their contraceptive utilization.    332 associated young women contraceptive utilization. Moreover, the more young women believe in  333 contraceptive myths was negatively associated with their contraceptive utilization.    338 DHS reports in Ethiopia [49, 50]. Moreover, our finding is far below 40.7% of Oromia; the region  339 where this study conducted but higher than3.4% of neighboring Somali regions’ level of utilization    338 DHS reports in Ethiopia [49, 50]. Moreover, our finding is far below 40.7% of Oromia, the region  339 where this study conducted, but higher than3.4% of neighboring Somali regions’ level of utilization    341 close cultural and religious similarities with the Somali region’s population, the region where the  342 contraceptive prevalence among the lowest in Ethiopia.    341 close cultural and religious similarities with the Somali region’s population, a region with a  342 contraceptive prevalence among the lowest in Ethiopia.    346 income countries [35, 51]. For instance, only 2% of contraceptive users were ever-mother 346 income countries [35, 51]. For instance, only 2% of contraceptive users were not-mothers (I ASSUME THIS IS AN ERROR)    347 likelihood of contraceptive  347 likelihood of contraception      351 marriage to delay the age of marriage  351 marriage to delay the age at marriage    366 mainly from husbands and mother in-law. Furthermore, evidence also showed that young women’s  366 mainly from husbands and mother in-law. Furthermore, evidence also showed young women’s    368 low contraceptive use in our study could be due to many of them comply with the social    368 low contraceptive use in our study could be due to many of them complying with the social    369 expectation which encourages childbearing and disapprove of contraception among young women.  369 expectation encouraging childbearing and disapproving of contraception among young women.    378 finding suggested that women can learn or receive support from friendships regarding  378 finding suggested that women can learn or receive support from friends regarding    388 myths showed the lower the likelihood of reporting contraceptive use  388 myths showed the lower likelihood of reporting contraceptive use    389 previous evidence that identified the negative effect contraceptive related misconceptions on  389 previous evidence that identified a negative effect of contraceptive related misconceptions on    413 approval of desired behavior (i.e., perceived social approval), beliefs of what would happen if fail  413 approval of desired behavior (i.e., perceived social approval), beliefs of what would happen if failing    417 of the social norm, understand the relations between the various constellations, and design tailored  417 of the social norm, understanding the relations between the various constellations, and designing tailored    426 myths and misconceptions about the modern contraceptive method.  426 myths and misconceptions regarding modern contraceptive methods    CHECK REFERENCES. Some include obvious errors:  446 2. CSA and ICF, Ethiopia Demographic and Health Sruvey 2016. 2017, Ethiopiam Central  447 Statistical Agency and ICF International: Addis Ababa, Ethiopia.    455 6. Guttmacher, I. Adding it up:Investing in Contraception and Maternal and Newborn Health for  456 Adolescents in Ethiopia, 2018, Fact Sheet, New York: Guttmacher Institute, 2017,  457 [https://www.guttmacher.org/fact-sheet/adding-it-up-contraception-mnh-2017. 2018](https://www.guttmacher.org/fact-sheet/adding-it-up-contraception-mnh-2017.%202018).    477 14. Yadav, D. and P. Dhillon, Assessing the impact of family planning advice on unmet need  478 and contraceptive use among currently married women in Uttar Pradesh, India. PloS one,  479 2015. 10(3): p. e0118584-e0118584.  477 14. Yadav, D. and P. Dhillon, Assessing the impact of family planning advice on unmet need  478 and contraceptive use among currently married women in Uttar Pradesh, India. PLOS ONE,  479 2015. 10(3): p. e0118584-e0118584.    506 24. UNICEF, What are Social Norms? How are They Measured? UNICEF  507 / UCSD Center on Global Justice  508 Project Cooperation Agreement, WORKING PAPER, M. Gerry and M. Francesca,  509 Editors. 2014 Sandiago, USA  (No dates???)    510 25. UKaid, Social norms, gender norms and adolescent girls: a brief guide. 2015.    513 27. Mardi, A., et al., Factors influencing the use of contraceptives through the lens of teenage  514 women: a qualitative study in Iran. 2018. 18(1): p. 202  JOURNAL, PP?    515 28. Kate, P. and J. Nicola Social norms stop Ethiopian girls from making safe choices about  516 pregnancy (unpublished). 2020.  LINK  517 F.N. and A.R. Veloso. Factors that influence the use of birth control by  518 Brazilian adolescents. in BBR Conference. 2012. Paulo – SP – Brazil.    534 35. Mutumba, M., E. Wekesa, and R. Stephenson, Community influences on modern  535 contraceptive use among young women in low and middle-income countries: a cross-sectional  536 multi-country analysis. BMC Public Health, 2018. 18(1): p. 430.  PAGES    587 56. Beyeza-Kashesya, J., et al., "Not a Boy, Not a Child": A Qualitative Study on Young  588 People's Views on Childbearing in Uganda. African Journal of Reproductive Health / La  589 Revue Africaine de la Sant&#xe9; Reproductive, 2010. 14(1): p. 71-81.    594 **S1 Table. Item used for**  594 **S1 Table. Items used for**   598 **S1 Minimal data set**  598 **S3 Minimal data set** | We have revised it based on your comments, and suggestion throughout the revised manuscript. |
