# Supplementary material for: Low contraceptive utilization among young married women is associated with perceived social norms and belief in contraceptive myths in rural Ethiopia
Source: PLoS One. 2021 Feb 22;16(2):e0247484. doi: 10.1371/journal.pone.0247484 (PMC7899365; doi:10.1371/journal.pone.0247484)
Supplement: Attachment — Submitted filename: Response to Reviewers.docx [file pone.0247484.s011.docx]

February 4, 2021

José Antonio Ortega, Ph.D.

Academic Editor-PLOS ONE

Re: Manuscript ID No. PONE-D-20-05351R2

Please find response to each point raised by the academic editor and/or reviewers, and a revised version of our manuscript “Low contraceptive utilization among young married women is associated with perceived social norms and belief in contraceptive myths in rural Ethiopia”, which we would like to resubmit for publication as an original article in PLOS ONE.

Your comments and those of the reviewers were highly insightful and enabled us to greatly improve the quality of our manuscript. Our point-by-point response to each of the point raised is attached with this letter. We hope that the revisions in the manuscript and our accompanying responses will be sufficient to make our manuscript suitable for publication in the PLOS ONE.

We shall look forward to hearing from you at your earliest convenience.

Yours sincerely,

**Title:** **Response to Reviewers comment of the Manuscript entitled “Low contraceptive utilization among young married women is associated with perceived social norms and belief in contraceptive myths in rural Ethiopia”**

| **Reviewer/Editor comments** | **Authors Response** |
| --- | --- |
| Despite the increasingly wider availability of contraception and the level of needs  33 in rural Ethiopia,  I asume you mean “Despite the increasingly wider availability of contraceptives and the high levels of potential demand [or unmet need] for family planning in rural Ethiopia, …” | Yes, we mean as you assumed. Thank you for your understanding! Now is corrected, line 32 and 33 of the revised manuscript |
| Substantial proportion of young married women in low and middle-income countries are at risk of  73 unintended pregnancy due to low contraceptive utilization than their adult counterparts. Young  74 women in the region also often used short-term methods that related to high failure and  75 discontinuation (8, 9).  The sentence is not grammatically or factually correct: what does the “than” mean, what is the comparison? As I said in the previous revision, what matters here is unmet need, and the levels are not highest among young married women. Also, women over 18 are adults. As it was discussed it is not true that levels of unmet need are highest. The second sentence is also missing a verb (I guess *are* related to higher ..). | We have revised the sentences in the revised manuscript, line 72 and 73 |
| Change proposed to old line 89-93 only partially adopted (missing the second part), and it now reads funny. | We apologize for missing the proposed change in the previous version. Now it is corrected on line 92 and 93 according to the prosed change during the previous comment or comment given on line 89-93 of the old version. |
| New line 102: studies have shown, not “studies have been shown” | Corrected in line 99 |
| New line 203: higher score indicates high believe in the myths or had 204 misconception about contraceptive methods.  Should be “a higher score indicates strong belief in the myths or misconceptions regarding contraceptive methods.” | Corrected according to your suggestion, line 196 and 917 |
| 216 or sure they were they could use contraceptive and  216 or sure they were they could use contraceptives and | Corrected in line 207 |
| 221 … she could use family planning even if her husband did not want. The items responses  221 … she could use family planning even if her husband did not want. The item responses | Corrected in line 2011 |
| 224 by the number of item (  224 by the number of items ( | Corrected in line 2013 |
| 231 The item response option  The item response options | Corrected in line 221 |
| 252 Exposure to FP information in last 12 months  252 Exposure to FP information in the last 12 months | Corrected in line 240 |
| 323 utilization **(**AOR= 0.60; 95% CI: 0.49-0.73). Moreover, odd of contraceptive utilization  323 utilization **(**AOR= 0.60; 95% CI: 0.49-0.73). Moreover, odds of contraceptive utilization | Corrected in line 306 |
| In several places, including table 1 and mention of variables: replace “Number of alive children” with “Number of children alive” | Corrected to in table1, table 2, and line 203, 254 |
| Also in this context “Had no children” replace with “Has no children” or, even better “No children alive”. | Corrected to ‘No children alive’ in table1 |
| Also in this context, replace “Three and above children” with “Three and more children” | Corrected to in table1 |
| 348 where this study conducted, but higher than 3.4% of neighboring Somali regions’ level of  349 utilization among reproductive-age women (2).  348 where this study was conducted, but higher than 3.4% in the neighboring Somali region (2). | Corrected according to your suggestion in line 330 |
| 396 with current contraceptive utilization. The more young women belief in contraceptive related  397 myths showed the lower the likelihood of reporting contraceptive use.   396 with current contraceptive utilization. The more young women believe in contraceptive related  397 myths, the lower the likelihood of reporting contraceptive use. | Corrected according to your suggestion on line 375 and 376, |
| 398 previous evidence that identified a negative effect contraceptive related  398 previous evidence that identified a negative effect of contraceptive related | Corrected in line 377 |
| 434 myths and misconceptions regarding the modern contraceptive methods  434 myths and misconceptions regarding modern contraceptive methods | Corrected in line 415 |
